# Association of serum ferritin and all-cause mortality in AKI patients: a retrospective cohort study

**DOI:** 10.3389/fmed.2024.1368719

**Published:** 2024-06-13

**Authors:** Xiaoxu Ren, Zhiming Jiang, Fen Liu, Quanzhen Wang, Hairong Chen, Lifeng Yu, Chaoqun Ma, Rong Wang

**Affiliations:** ^1^Department of Nephrology, Shandong Provincial Hospital, Shandong University, Jinan, Shandong, China; ^2^Department of Critical Care Medicine, The First Affiliated Hospital of Shandong First Medical University & Shandong Provincial Qianfoshan Hospital, Shandong medicine and Health Key Laboratory of Emergency Medicine, Shandong Institute of Anesthesia and Respiratory Critical Medicine, Jinan, Shandong, China; ^3^Department of Emergency Medicine, Shandong Provincial Hospital Affiliated to Shandong First Medical University, Jinan, Shandong, China

**Keywords:** serum ferritin, hyperferritinemia, acute kidney injury, critical ill patient, mortality

## Abstract

**Background:**

Serum ferritin (SF) is clinically found to be elevated in many disease conditions, and our research examines serum ferritin in patients with acute kidney injury (AKI) and its implication on the risk of short-term mortality in AKI.

**Methods:**

Data were extracted from the Medical Information Mart for Intensive Care IV 2.2 (MIMIC-IV 2.2) database. Adult patients with AKI who had serum ferritin tested on the first day of ICU admission were included. The primary outcome was 28-day mortality. Kaplan–Meier survival curves and Cox proportional hazards models were used to test the relationship between SF and clinical outcomes. Subgroup analyses based on the Cox model were further conducted.

**Results:**

Kaplan–Meier survival curves showed that a higher SF value was significantly associated with an enhanced risk of 28-day mortality, 90-day mortality, ICU mortality and hospital mortality (log-rank test: *p* < 0.001 for all clinical outcomes). In multivariate Cox regression analysis, high level of SF with mortality was significantly positive in all four outcome events (all *p* < 0.001). This result remains robust after adjusting for all variables. Subgroup analysis of SF with 28-day mortality based on Cox model-4 showed that high level of SF was associated with high risk of 28-day mortality in patients regardless of the presence or absence of sepsis (*p* for interaction = 0.730). Positive correlations of SF and 28-day mortality were confirmed in all other subgroups (*p* for interaction>0.05).

**Conclusion:**

High level of SF is an independent prognostic predictor of 28-day mortality in patients with AKI.

## 1 Introduction

Acute kidney injury (AKI) is defined by a rapid loss of kidney function, as evidenced by elevated blood creatinine and decreased urine output (1, 2). Severe AKI may lead to death, and even with proper fluid management and renal replacement therapy, a proportion of patients may still develop chronic kidney disease (CKD), resulting in decreased quality of life and a significant healthcare burden on society (1–3). As the blood creatinine and urine output indicate the loss of kidney filtration function, direct assessment of structural damage to the kidney is not available at the moment. Searching for kidney damage biomarkers is an endeavor of many researchers (4, 5).

Serum ferritin is often used clinically as an indicator of iron store, and it has been found to be elevated in inflammatory diseases (6, 7), malignancies (7, 8), coronary heart disease (9, 10), and other conditions (11). In clinical work, many researchers including us have noticed that serum ferritin is also elevated in AKI patients. However, there are arguments as to whether elevated serum ferritin is associated with the risk of adverse events (12–14). In this study, we collected data on critical ill patients with AKI to explore whether serum ferritin elevation is associated with adverse prognosis in patients with AKI.

## 2 Materials and methods

### 2.1 Source of data

This is a retrospective cohort study. All data were obtained from the Medical Information Mart for Intensive Care (latest version MIMIC-IV 2.2) database^1^. MIMIC is a public database and contains the clinical information of critical ill patients from 2008 to 2019 who admitted in intensive care unit (ICU) of Beth Israel Deaconess Medical Center (Boston, Massachusetts) (15). The author has passed the Protecting Human Research Participants exam and obtained the certification (certification number 38100618) for utilizing the MIMIC-IV database.

### 2.2 Study design

The definition of AKI was based on the KDIGO (Kidney Disease: Improving Global Outcomes) 2012 diagnostic criteria (16). Patients who met the criteria as follow were excluded: (1) < 18 years old; (2) not the first admission of ICU; (3) patients comorbid hematologic diseases, tumor, carcinoma, hemorrhage diseases, or undergoing maternity events; (4) missing data of serum ferritin; (5) exclude 2 cases with negative survival times (time to death minus time to admission). The final number of cases included in the study was 4,712.

Our manuscript was written in accordance with the STROBE statement guidelines (17).

### 2.3 Variables

Variables were extracted after admission and only first lab result in ICU of each variable was utilized. The following information and variables were extracted: age, gender, disease rating scores [eGFR (18), OASIS, SAPS ii, SOFA], comorbidities (Sepsis3, Myocardial Infarct, Congestive Heart Failure, Cerebrovascular Disease, Severe Liver Disease, Chronic Pulmonary Disease and Diabetes). Comorbidities extracts Charlson Comorbidity Index (CCI) (19, 20) based on the recorded ICD-9 and ICD-10 codes (21). For details, please check Supplementary files S2 (comorbidity ICD codes), treatments (renal replacement therapy (RRT), ventilation), length of stay (LOS) in ICU and hospital, survival time, lab events [serum ferritin (SF), serum creatinine, blood urea nitrogen (BUN), albumin, white blood cell (WBC), red blood cell (RBC), hemoglobin, platelets].

### 2.4 Outcomes

The primary study outcome was 28-day mortality, which best characterizes short-term survival in patients with AKI. Other findings include 90-day mortality, hospital mortality, and ICU mortality to further illustrate the relationship between SF and the risk of death from AKI.

### 2.5 Management of abnormal values and missing data

The indicators with more than 25% missing values were removed from the final analysis. We had planned to include C-reactive protein and ESR (erythrocyte sedimentation rate), two acute-phase reactive proteins, as a comparison of SF, and cystatin C as an indicator of renal injury in our study, but these three variables were excluded because they had more than 50% missing values in the study population. The detail of the missing value is shown in Supplementary files S1 (final cohort).

### 2.6 Statistical analysis

For analysis, we grouped SF in thirds according to percentile. For continuous quantitative data, if it conformed to normal distribution, it was described by mean ± standard deviation, and one-way ANOVA was used for comparison between three groups; if it did not conform to normal distribution, it was statistically described by median [P25, P75], and rank-sum test was used for comparison between groups; for counting data, it was described by number of cases (%), and chi-square test or Fisher’s exact probability method was used for comparison between groups.

To explore the association between SF and death, Kaplan–Meier survival curves were performed and survival curves were plotted to test the association between different SF levels and death, and the survival curves were compared using the log rank test. Because of the high dispersion of SF value, we performed a log transformation in the subsequent analysis.

Further multivariate Cox regression was used to explore the association between SF and death, hazard ratio and 95% confidence interval were calculated, and different covariates were corrected in multivariate regression analysis.

To further explore the association of SF with death, restricted cubic spline plots were plotted using the R language rms package to explore the association of SF values with death and to correct for the relevant covariates (as in Cox model-4).

To explore whether the association between SF and death varied by different characteristics, subgroup analyses were performed and interaction effects were tested by likelihood ratio tests.

All statistical analyses and related graphing were performed in R software (version 4.3.2), and two-sided *p* < 0.05 was considered statistically significant.

### 2.7 Sensitivity analysis

Patients with comorbid hematologic diseases, tumor, carcinoma, hemorrhage diseases, or undergoing maternity events were excluded. The study population was then subgroup analyzed according to whether or not they were comorbid with sepsis, myocardial infarct, congestive heart failure, cerebrovascular disease, severe liver disease, chronic pulmory disease and diabetes.

Due to the large dispersion of SF values, we performed a logarithmic transformation in subsequent analysis. Cox and subgroup analyses were done with SF as a continuous and categorical variable, respectively.

## 3 Results

### 3.1 Patients selection

**Figure 1:**
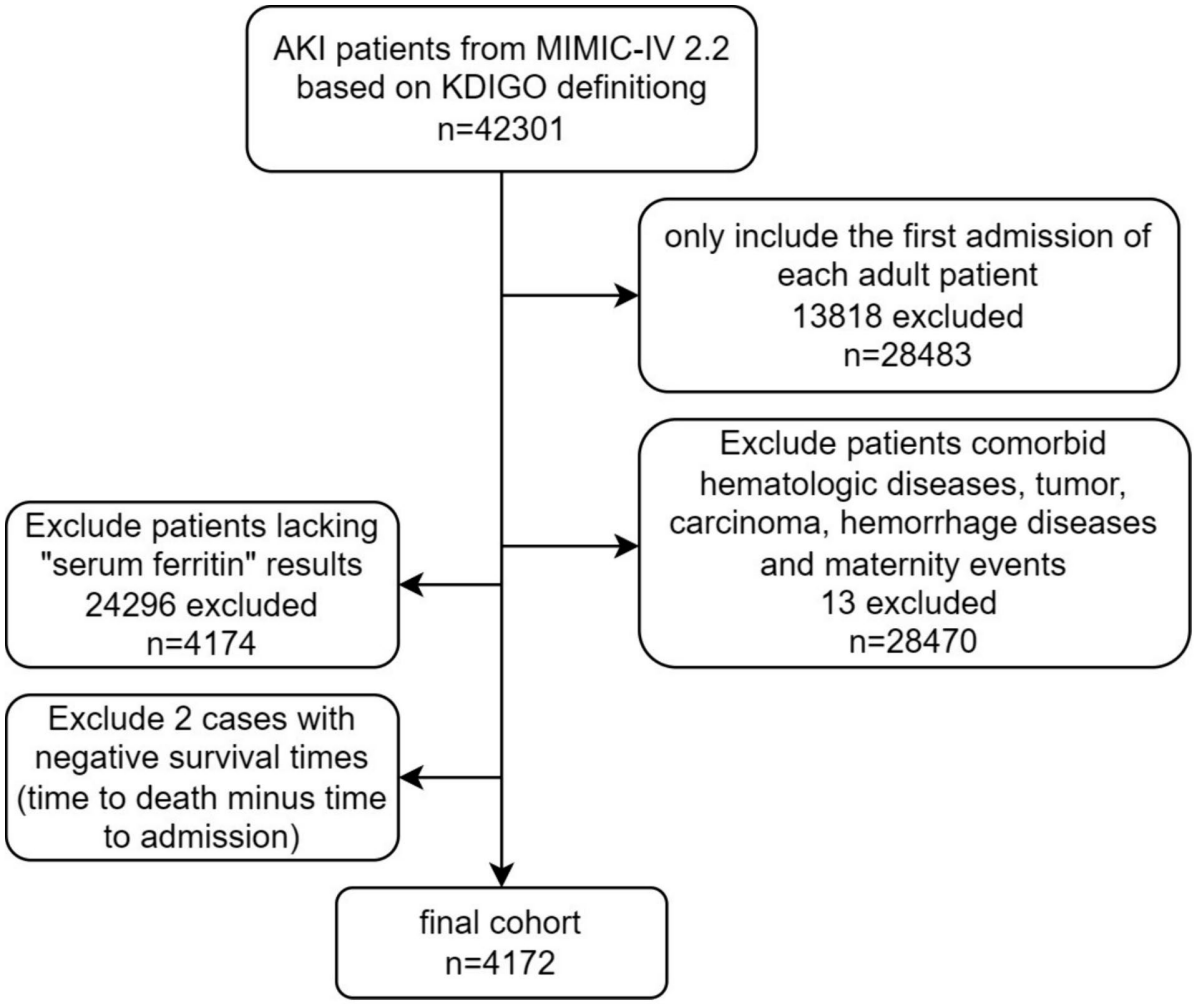
Study design flowchart.

There were 4,712 AKI patients included in the final analysis. Figure 1 presents the process of patients’ selection process.

### 3.2 Patient demographics and baseline characteristics

As shown in Table 1, patients were subdivided into 3 groups based on SF value percentile (Q1 [4.2, 250] ng/mL, Q2 [250,680] ng/mL, Q3 [680, 200,205] ng/mL). According to the normal range of SF values given in the MIMIC-IV 2.2 database (30–400 ng/mL for males and 13–150 for females), it can be noticed that SF was mostly at the normal range for patients in group Q1 and at the high level for patients in Q2 and Q3. Patients in the Q2 and Q3 had a higher percentage of stage 3-AKI (29.4% in Q1, 36.1% in Q2, 49.5% in Q3). However, in terms of eGFR (estimated glomerular filtration rate, an indicator of renal filtration function), the mean eGFR was rather lower in group Q1 (0.92 ± 0.13 in Q1, 0.95 ± 0.13 in Q2, 0.97 ± 0.13 in Q3). In groups Q2 and Q3, in addition to higher SF values, these patients had higher mean disease severity assessment scores (OASIS, SAPSii, SOFA) than group Q1. Similar elevated trends were seen across outcome events such as length of stay in ICU (LOS-ICU), length of stay in hospital (LOS-hospital), 28-day mortality, 90-day mortality, hospital mortality and ICU mortality, demonstrated longer ICU or hospital stays and higher mortality rates in the Q2 and Q3 groups. As for the laboratory test results, the difference in hemoglobin between the three groups was not significant (*p* = 0.051). The mean values of creatinine and BUN were higher in groups Q2 and Q3 than in group Q1, while the mean values of red blood cells and platelets were lower than in group Q1. Similar to previous studies (22), the proportion of patients with sepsis was relatively higher in the Q2 and Q3 groups, which had higher SF values.

**Table 1:** Patient demographics and baseline characteristics.

Variables	Total (*n* = 4,172)	SF groups			*p-*value
		Q1 (*n* = 1,392)	Q2 (*n* = 1,393)	Q3 (*n* = 1,387)	
Age, Mean ± SD (year)	65.08 ± 16.69	66.99 ± 17.03	65.74 ± 16.48	62.50 ± 16.24	< 0.001
Gender, *n* (%)					< 0.001
Female	1969 (47.2)	790 (56.8)	618 (44.4)	561 (40.4)	
Male	2,203 (52.8)	602 (43.2)	775 (55.6)	826 (59.6)	
AKI stage, *n* (%)					< 0.001
1	920 (22.1)	360 (25.9)	274 (19.7)	286 (20.6)	
2	1,653 (39.6)	623 (44.8)	616 (44.2)	414 (29.8)	
3	1,599 (38.3)	409 (29.4)	503 (36.1)	687 (49.5)	
Sepsis3, *n* (%)					< 0.001
No	1,222 (29.3)	540 (38.8)	387 (27.8)	295 (21.3)	
Yes	2,950 (70.7)	852 (61.2)	1,006 (72.2)	1,092 (78.7)	
Myocardial Infarct, *n* (%)					0.190
No	3,388 (81.2)	1,119 (80.4)	1,121 (80.5)	1,148 (82.8)	
Yes	784 (18.8)	273 (19.6)	272 (19.5)	239 (17.2)	
Congestive Heart Failure, *n* (%)					< 0.001
No	2,587 (62)	743 (53.4)	885 (63.5)	959 (69.1)	
Yes	1,585 (38)	649 (46.6)	508 (36.5)	428 (30.9)	
Cerebrovascular Disease, *n* (%)					< 0.001
No	3,645 (87.4)	1,180 (84.8)	1,226 (88)	1,239 (89.3)	
Yes	527 (12.6)	212 (15.2)	167 (12)	148 (10.7)	
Severe Liver Disease, *n* (%)					0.002
No	3,724 (89.3)	1,262 (90.7)	1,257 (90.2)	1,205 (86.9)	
Yes	448 (10.7)	130 (9.3)	136 (9.8)	182 (13.1)	
Chronic Pulmory Disease, *n* (%)					< 0.001
No	2,977 (71.4)	932 (67)	1,006 (72.2)	1,039 (74.9)	
Yes	1,195 (28.6)	460 (33)	387 (27.8)	348 (25.1)	
Diabetes, *n* (%)					< 0.001
No	2,744 (65.8)	852 (61.2)	908 (65.2)	984 (70.9)	
Yes	1,428 (34.2)	540 (38.8)	485 (34.8)	403 (29.1)	
RRT, *n* (%)					< 0.001
No	3,462 (83)	1,296 (93.1)	1,187 (85.2)	979 (70.6)	
Yes	710 (17)	96 (6.9)	206 (14.8)	408 (29.4)	
Ventilation, *n* (%)					0.159
No	501 (12)	185 (13.3)	164 (11.8)	152 (11)	
Yes	3,671 (88)	1,207 (86.7)	1,229 (88.2)	1,235 (89)	
eGFR, Mean ± SD (ml/min/1.73m^2^)	0.95 ± 0.13	0.92 ± 0.13	0.95 ± 0.13	0.97 ± 0.13	< 0.001
OASIS, Mean ± SD	34.09 ± 8.72	32.56 ± 8.16	33.98 ± 8.62	35.75 ± 9.08	< 0.001
SAPSii, Mean ± SD	40.92 ± 14.29	37.63 ± 13.14	40.31 ± 13.59	44.83 ± 15.14	< 0.001
SOFA score, Mean ± SD	3.89 ± 2.19	3.34 ± 1.75	3.81 ± 2.06	4.51 ± 2.55	< 0.001
Creatinine, Mean ± SD (mg/dl)	2.10 ± 2.47	1.66 ± 1.69	2.06 ± 2.57	2.57 ± 2.90	< 0.001
BUN, Mean ± SD (mg/dl)	35.70 ± 29.57	32.38 ± 26.86	35.56 ± 30.02	39.19 ± 31.27	< 0.001
Albumin, Mean ± SD (mg/dl)	2.96 ± 0.64	3.13 ± 0.60	2.93 ± 0.63	2.83 ± 0.65	< 0.001
WBC, Mean ± SD (10^9/L)	12.49 ± 10.75	11.60 ± 9.41	12.20 ± 7.06	13.69 ± 14.38	< 0.001
RBC, Mean ± SD (10^9/L)	3.47 ± 0.81	3.60 ± 0.78	3.48 ± 0.80	3.35 ± 0.83	< 0.001
Hemoglobin, Mean ± SD (mg/dl)	10.25 ± 2.28	10.16 ± 2.18	10.37 ± 2.23	10.23 ± 2.43	0.051
Platelets, Mean ± SD (10^12/L)	213.00 ± 126.87	232.34 ± 120.83	214.96 ± 130.56	191.62 ± 125.83	< 0.001
LOS-ICU, Mean ± SD (day)	7.20 ± 8.87	5.76 ± 7.11	7.09 ± 8.25	8.74 ± 10.66	< 0.001
LOS-Hospital, Mean ± SD (day)	18.94 ± 18.21	15.35 ± 14.68	18.80 ± 16.61	22.68 ± 21.84	< 0.001
28-day mortality, *n* (%)					< 0.001
Survival	3,509 (84.1)	1,234 (88.6)	1,205 (86.5)	1,070 (77.1)	
Non survival	663 (15.9)	158 (11.4)	188 (13.5)	317 (22.9)	
90-day mortality, *n* (%)					< 0.001
Survival	3,099 (74.3)	1,110 (79.7)	1,078 (77.4)	911 (65.7)	
Non survival	1,073 (25.7)	282 (20.3)	315 (22.6)	476 (34.3)	
Hospital mortality, *n* (%)					< 0.001
survival	3,589 (86)	1,274 (91.5)	1,238 (88.9)	1,077 (77.6)	
Non survival	583 (14)	118 (8.5)	155 (11.1)	310 (22.4)	
ICU mortality, *n* (%)					< 0.001
Survival	3,824 (91.7)	1,325 (95.2)	1,309 (94)	1,190 (85.8)	
Non survival	348 (8.3)	67 (4.8)	84 (6)	197 (14.2)	

Patients were subdivided into 3 groups according to SF value percentile ([4.2, 250] Q1, [250,680] Q2, [680, 200,205] Q3), and the baseline characteristics and outcomes were further compared among those three groups. Variable was described by mean ± standard deviation, and one-way ANOVA was used for comparison between three groups; if it did not conform to normal distribution, it was statistically described by median [P25, P75], and rank-sum test was used for comparison between groups; for counting data, it was described by number of cases (%), and chi-square test or Fisher’s exact probability method was used for comparison between groups.

AKI, acute kidney injury; SF, serum ferritin; LOS, length of stay; eGFR, estimated glomerular filtration rate; OASIS, Oxford acute severity of illness score; SAPS ii, Simplified Acute Physiology Score ii; SOFA, Sequential Organ Failure Assessment; RRT, renal replacement treatment; WBC, white blood cell; RBC, red blood cell, BUN, the blood urea nitrogen.

We estimate the eGFR using Simplified MDRD: eGFR = 186 * Scr ^ (−1.154) * Age ^ (−0.203) * 0.742 Female.

### 3.3 SF and mortality

**Figure 2:**
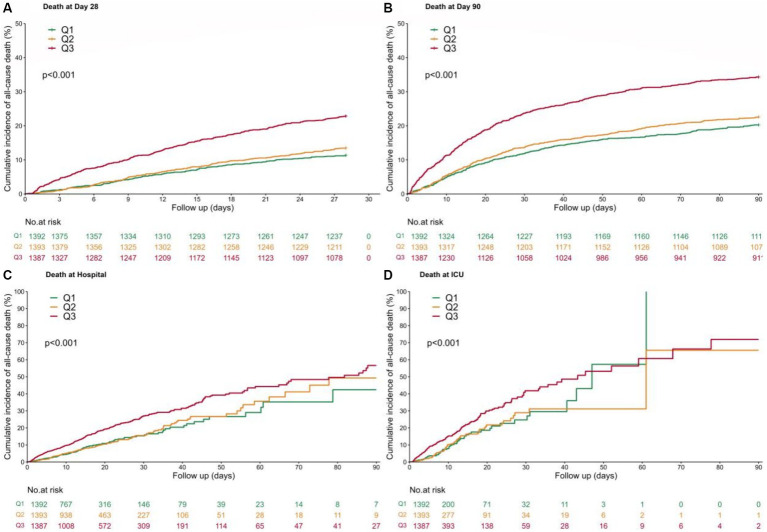
Kaplan–Meier curves. Patients were subdivided into 3 groups according to SF value percentile ([4.2, 250] Q1, [250,680] Q2, [680, 200,205] Q3), with a follow-up period of 90 days. **(A)** 28-day mortality; **(B)** 90-day mortality; **(C)** Hospital mortality; **(D)** ICU mortality.

Kaplan–Meier analysis curves demonstrated that a higher SF value was significantly associated with an enhanced risk of ICU mortality, hospital mortality, 28-day mortality and 90-day mortality (log-rank test: *p* < 0.001 for all clinical outcomes) (Figure 2).

**Figure 3:**
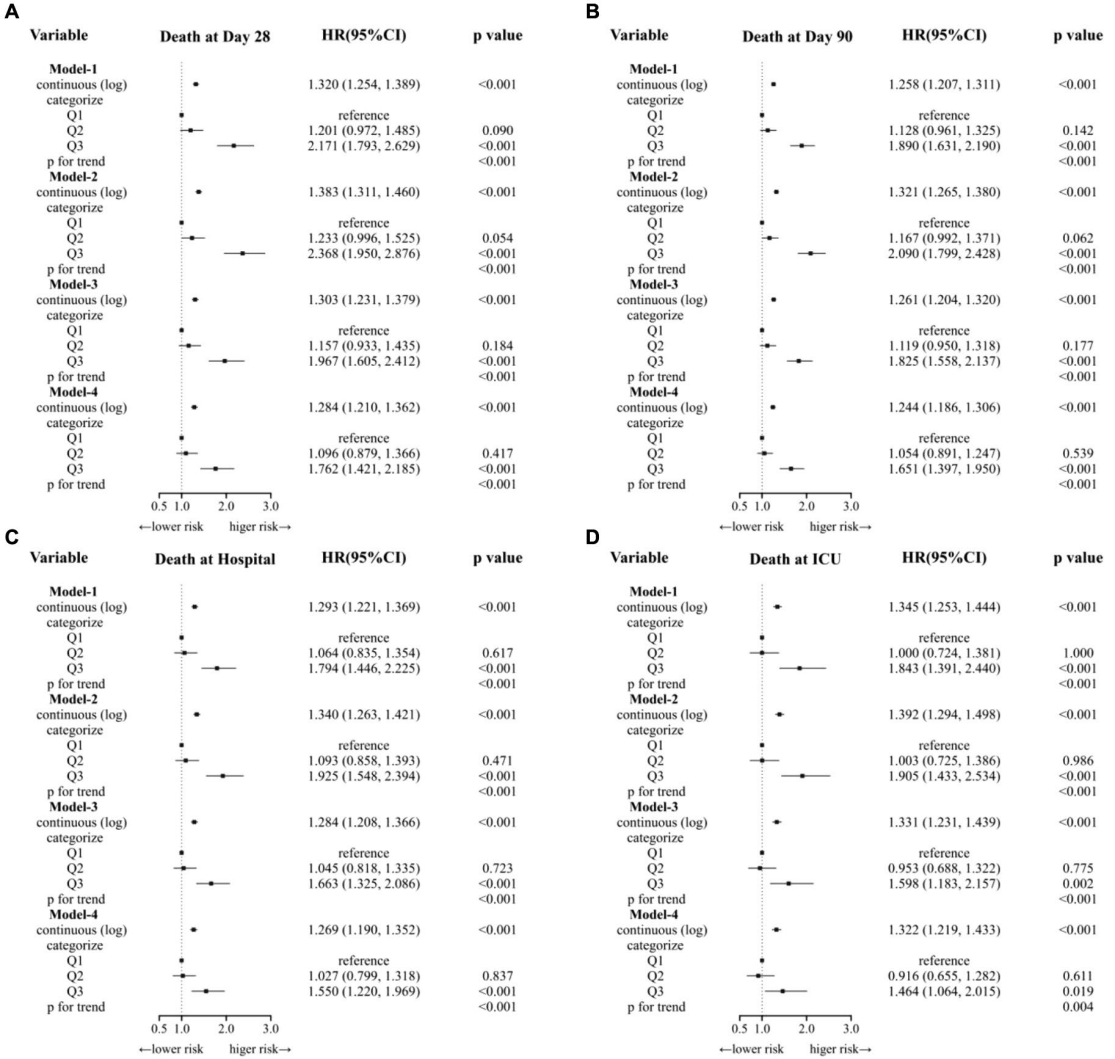
Cox regression models to analysis the association of SF and clinical outcomes. **(A)** Model-1 was the crude model. **(B)** Model-2 adjusted for age, gender. **(C)** Model-3 adjusted for Model-2 + AKI stage, Sepsis3, Myocardial Infarct, Congestive Heart Failure Cerebrovascular Disease, Severe Liver Disease, Chronic Pulmory Disease, Diabetes, RRT, Ventilation. **(D)** Model-4 adjusted for Model-3 + creatine, BUN, albumin, WBC, RBC, hemoglobin, platelets.

We conducted Cox regression models to analysis the association of SF and clinical outcomes (Table 2 and Figure 3). When SF was used as a continuous variable, its relationship with mortality was significantly positive in all four outcome events. This result remains robust after adjusting for all variables (model-4). When considered as a categorical variable, the Q1 group was used as a reference, Q3 group ((680, 200,205) ng/mL) was associated with a higher risk of mortality in all four outcome events. Meanwhile, the Q2 group ((250,680) ng/mL) did not show a significantly higher HR. In all four models, HR values were consistently and significantly higher for the Q3 group.

**Table 2:** Cox regression models to analysis the association of SF and clinical outcomes.

Variable	HR (95% CI)	*p*-value	Variable	HR (95% CI)	*p*-value
28-day Mortality			Hospital Mortality		
Model-1			Model-1		
continuous (log)	1.320 (1.254, 1.389)	<0.001	continuous (log)	1.293 (1.221, 1.369)	<0.001
categorize			categorize		
Q1	reference		Q1	reference	
Q2	1.201 (0.972, 1.485)	0.090	Q2	1.064 (0.835, 1.354)	0.617
Q3	2.171 (1.793, 2.629)	<0.001	Q3	1.794 (1.446, 2.225)	<0.001
*p* for trend		<0.001	*p* for trend		<0.001
Model-2			Model-2		
continuous (log)	1.383 (1.311, 1.460)	<0.001	continuous (log)	1.340 (1.263, 1.421)	<0.001
categorize			categorize		
Q1	reference		Q1	reference	
Q2	1.233 (0.996, 1.525)	0.054	Q2	1.093 (0.858, 1.393)	0.471
Q3	2.368 (1.950, 2.876)	<0.001	Q3	1.925 (1.548, 2.394)	<0.001
*p* for trend		<0.001	*p* for trend		<0.001
Model-3			Model-3		
continuous (log)	1.303 (1.231, 1.379)	<0.001	continuous (log)	1.284 (1.208, 1.366)	<0.001
categorize			categorize		
Q1	reference		Q1	reference	
Q2	1.157 (0.933, 1.435)	0.184	Q2	1.045 (0.818, 1.335)	0.723
Q3	1.967 (1.605, 2.412)	<0.001	Q3	1.663 (1.325, 2.086)	<0.001
*p* for trend		<0.001	*p* for trend		<0.001
Model-4			Model-4		
continuous (log)	1.284 (1.210, 1.362)	<0.001	continuous (log)	1.269 (1.190, 1.352)	<0.001
categorize			categorize		
Q1	reference		Q1	reference	
Q2	1.096 (0.879, 1.366)	0.417	Q2	1.027 (0.799, 1.318)	0.837
Q3	1.762 (1.421, 2.185)	<0.001	Q3	1.550 (1.220, 1.969)	<0.001
*p* for trend		<0.001	*p* for trend		<0.001
90-day mortality			ICU Mortality		
Model-1			Model-1		
continuous (log)	1.258 (1.207, 1.311)	<0.001	continuous (log)	1.345 (1.253, 1.444)	<0.001
categorize			categorize		
Q1	reference		Q1	reference	
Q2	1.128 (0.961, 1.325)	0.142	Q2	1.000 (0.724, 1.381)	1.000
Q3	1.890 (1.631, 2.190)	<0.001	Q3	1.843 (1.391, 2.440)	<0.001
*p* for trend		<0.001	*p* for trend		<0.001
Model-2			Model-2		
continuous (log)	1.321 (1.265, 1.380)	<0.001	continuous (log)	1.392 (1.294, 1.498)	<0.001
categorize			categorize		
Q1	reference		Q1	reference	
Q2	1.167 (0.992, 1.371)	0.062	Q2	1.003 (0.725, 1.386)	0.986
Q3	2.090 (1.799, 2.428)	<0.001	Q3	1.905 (1.433, 2.534)	<0.001
*p* for trend		<0.001	*p* for trend		<0.001
Model-3			Model-3		
continuous (log)	1.261 (1.204, 1.320)	<0.001	continuous (log)	1.331 (1.231, 1.439)	<0.001
categorize			categorize		
Q1	reference		Q1	reference	
Q2	1.119 (0.950, 1.318)	0.177	Q2	0.953 (0.688, 1.322)	0.775
Q3	1.825 (1.558, 2.137)	<0.001	Q3	1.598 (1.183, 2.157)	0.002
*p* for trend		<0.001	*p* for trend		<0.001
Model-4			Model-4		
continuous (log)	1.244 (1.186, 1.306)	<0.001	continuous (log)	1.322 (1.219, 1.433)	<0.001
categorize			categorize		
Q1	reference		Q1	reference	
Q2	1.054 (0.891, 1.247)	0.539	Q2	0.916 (0.655, 1.282)	0.611
Q3	1.651 (1.397, 1.950)	<0.001	Q3	1.464 (1.064, 2.015)	0.019
*p* for trend		<0.001	*p* for trend		0.004

Model-1 was the crude model.

Model-2 adjusted for age, gender.

Model-3 adjusted for Model-2 + AKI stage, Sepsis3, Myocardial Infarct, Congestive Heart FailurCerebrovascular Disease, Severe Liver Disease, Chronic Pulmory Disease, Diabetes, RRT, Ventilation.

Model-4 adjusted for Model-3 + creatine, BUN, albumin, WBC, RBC, hemoglobin, platelets.

**Figure 4:**
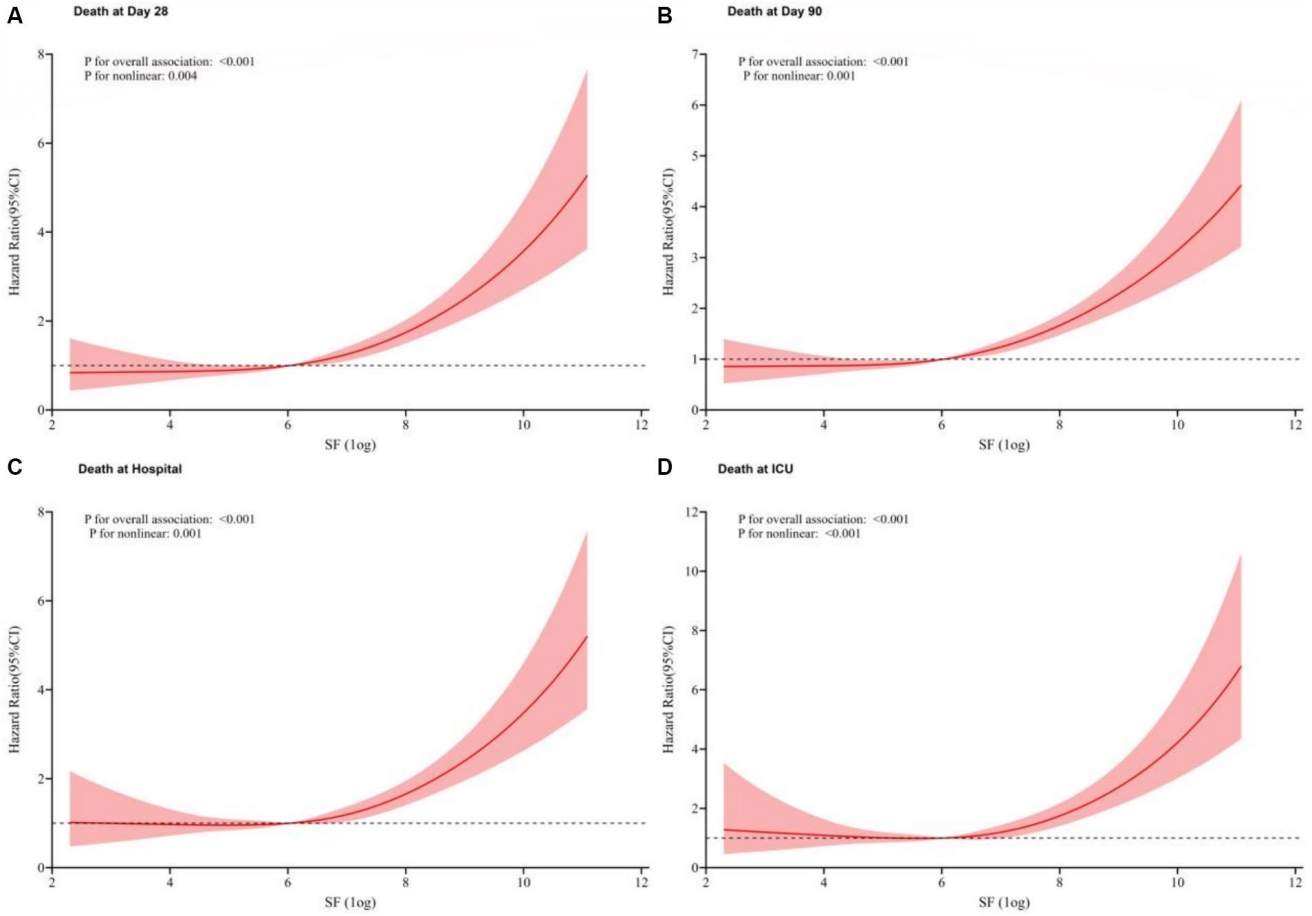
Restricted cubic spline models based on Cox modle-4. Due to the large dispersion of SF values, we performed a logarithmic transformation in subsequent analysis. The relationship of SF levels and all four outcomes were nonlinearly positive. **(A)** 28-day mortality; **(B)** 90-day mortality; **(C)** Hospital mortality; **(D)** ICU mortality.

Through multivariate Cox regression analysis and smooth curve fitting, we found that the relationship of SF levels and all four outcomes were nonlinear (Figure 4), and the higher the value of SF, the higher the HR for the occurrence of a mortality event (*p* for trend<0.001).

### 3.4 Subgroup analysis

**Figure 5:**
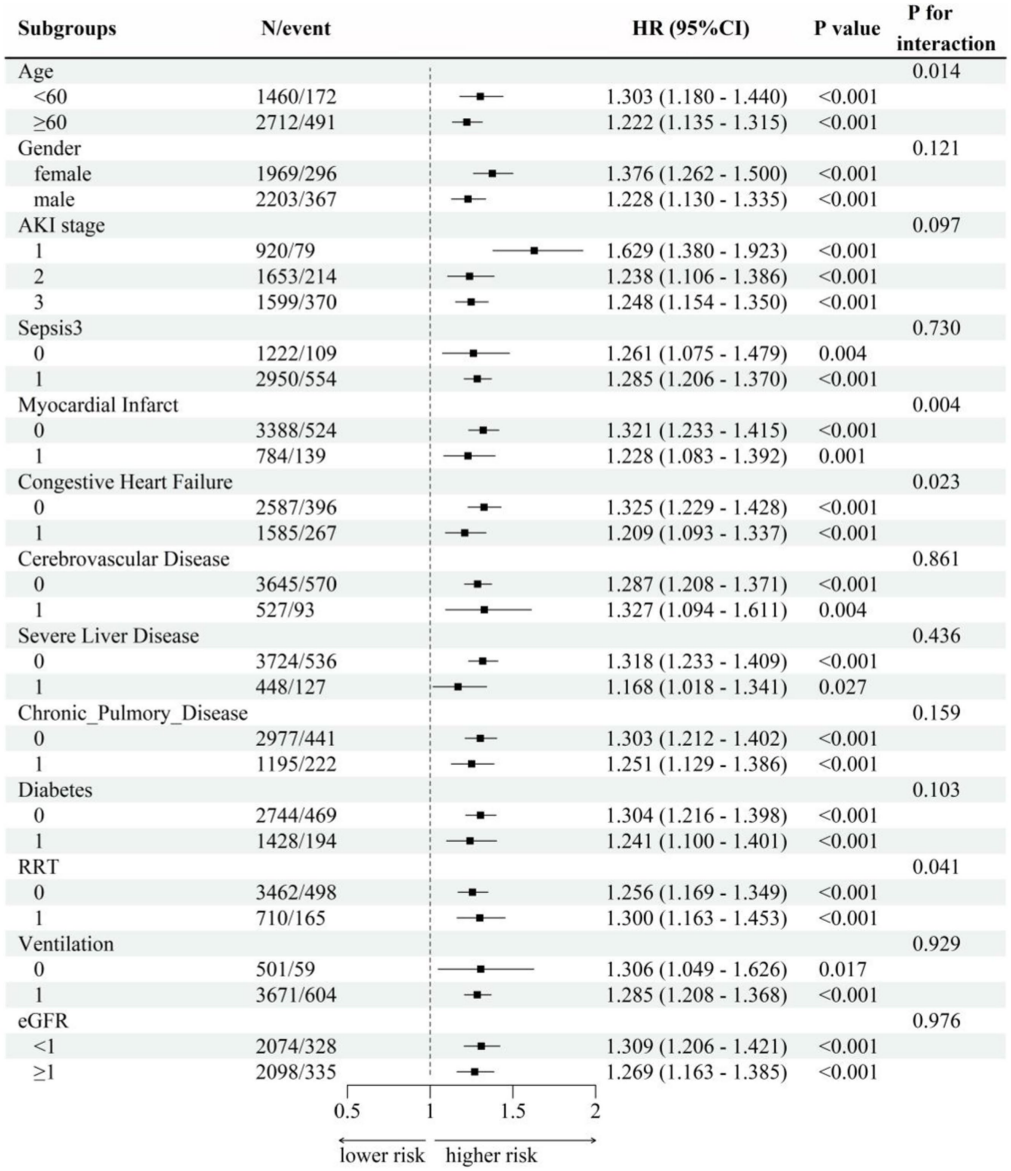
Subgroup analysis. Subgroup analyses were performed and interaction effects were tested by likelihood ratio tests to explore whether the association between SF and death varied by different characteristics. Except for the stratification factor itself, the stratifications were adjusted for all variables (age, gender, AKI stage, eGFR, OASIS, SAPS ii, SOFA, Sepsis3, Myocardial Infarct, Congestive Heart Failure, Cerebrovascular Disease, Severe Liver Disease, Chronic Pulmonary Disease, Diabetes, RRT, ventilation, creatinine, BUN, albumin, WBC, RBC, hemoglobin, platelets).

Figure 5 showed subgroup analysis results of SF (as a continuous variable) with the primary outcome 28-day mortality based on Cox model-4. Subgroup analyses were performed and interaction effects were tested by likelihood ratio tests to explore whether the association between SF and death varied by different characteristics. Except for the stratification factor itself, the stratifications were adjusted for all variables (age, gender, AKI stage, eGFR, OASIS, SAPS ii, SOFA, Sepsis3, Myocardial Infarct, Congestive Heart Failure, Cerebrovascular Disease, Severe Liver Disease, Chronic Pulmonary Disease, Diabetes, RRT, ventilation, creatinine, BUN, albumin, WBC, RBC, hemoglobin, platelets). Positive correlations of SF and 28-day mortality were confirmed in all other subgroups (p for interaction>0.05). High level of SF was associated with higher risk of 28-day mortality in patients <60 years old (HR = 1.303, 95% CI: 1.180–1.440), patients without myocardial infarct (HR = 1.321, 95% CI: 1.233–1.415), patients without congestive heart failure (HR = 1.325, 95% CI: 1.229–1.428) and patients used RRT (HR = 1.300, 95% CI: 1.163–1.453), all p for interaction <0.05. In the sepsis subgroup, which was of greater interest to us, we found a positive association between SF and 28-day mortality regardless of the presence or absence of sepsis (interaction *p* = 0.730).

## 4 Discussion

Acute kidney injury (AKI) is the sudden loss of excretory function of the kidneys and has a high prevalence in critically ill patients. As a global disease, the causes of AKI vary in different regions. Infection and trauma are the most common causes of AKI. In less developed regions, infection, and hypovolemic shock are the main causes of AKI. In developed countries, AKI occurs mostly in hospitalized elderly patients and is associated with sepsis, nephrotoxic drugs, or invasive procedures. The diverse etiology of AKI implies diverse pathophysiological mechanisms. AKI that occurs in ICU mostly belongs to one of the multiorgan failure categories and is often accompanied by hepatic impairment, respiratory failure, heart failure, and shock, which carries a higher risk of death (1, 16).

As shown in Table 1, Our study population was included AKI patients admitted to the ICU, most of whom had multi-organ dysfunction and required RRT (17%) and respiratory ventilation support (88%). 70.7% of these patients met the diagnostic criteria for Sepsis 3 (Third International Consensus Definitions for Sepsis and Septic Shock), 34.2% of them had comorbid diabetes mellitus, and some of them had comorbid cardiovascular and other diseases, whereas previous studies have shown that elevated serum ferritin may occur in these disease states. Previous studies have found that ferritin is not only an indicator of iron stores, but is also abnormally elevated in inflammatory responses, tumors, and other conditions. Studies of Sepsis (23), acute cardiac infarction (9), cerebrovascular disease (24), and rheumatoid disorders have suggested a correlation between elevated ferritin and poor disease prognosis (25, 26). Therefore, our study does not imply that elevated serum ferritin is a specific manifestation of AKI, but rather the result of a combination of systemic diseases in critically ill patients.

Most critically ill patients are in a state of high depletion and negative nitrogen balance, with impaired gastrointestinal function and low early energy and nutrient intake (27–29), resulting in the fact that traditional markers of kidney injury, such as serum creatinine and blood urea nitrogen, may be insufficient to comprehensively assess critically ill AKI patients. As an indicator of iron stores and acute phase response, serum ferritin can be a good addition to the assessment of the condition of patients with severe AKI. We suggest that there may be several reasons for elevated SF in AKI patients: (1) Infections and other conditions lead to an inflammatory response, which promotes ferritin synthesis (12, 30). (2) Tissue and cellular destruction lead to the release of intracellular iron into the bloodstream, resulting in the formation of more ferritin (7). (3) Disturbances in the internal environment might lead to impaired iron utilization and lower ferritin consumption (14, 31). All three of these causes are a result of the seriousness of the disease, and therefore the more serious the disease the higher SF is likely to be, which is accompanied by a worse prognosis. This is consistent with our findings. Most basic medical research on AKI has focused on renal tubular injury. Ferroptosis is a type of programmed cell death driven by iron-dependent phospholipid peroxidation. Recent studies have shown that ferroptosis is elevated in tubular cell ferroptosis (32, 33). However, ferroptosis as an intracellular phenomenon in relation to circulating iron has not been correlated and deserves continued attention in this direction.

In our study, we found that AKI patients in the ICU with elevated SF had a higher length of hospitalization, length of ICU stay and higher risk of death (28-day mortality, 90-day mortality, hospital mortality and ICU mortality) than other patients, and this high risk was still significant after applying fully adjustment. Grouping by tertiles showed that SF was generally in the normal range in group Q1, highest in group Q3, and HR for each mortality event was significantly higher in group Q3 compared to group Q1, whereas in group Q2, the increase in HR was not significant. Curve fitting showed that the HR for the occurrence of each mortality event was increasing as the SF was higher, and the value of SF was positively and curvilinearly correlated with the risk of death.

The predictive value of serum ferritin for all-cause mortality in critically ill and septic patients has been previously reported (22, 23). Ferritin levels were higher in septic patients than in patients with other diagnoses in the intensive care unit, and were higher in patients who developed septic shock (22). Our subgroup analysis revealed that AKI patients in the Q3 group (SF∈ (680, 200,205) ng/ml) had an increased risk of death within 28 days regardless of the presence of sepsis (sepsis: HR = 1.285 95% CI (1.206–1.370); without sepsis: HR = 1.261,95% CI (1.075–1.479); *p* for interaction = 0.730). We subgrouped age, gender, and common comorbidities, the HR for 28-day mortality was significantly higher in the Q3 group in all subgroups. This suggests that high SF (>680 ng/mL) is an independent risk factor for the occurrence of death within 28 days in patients with AKI.

A few previous studies have suggested that high ferritin is associated with a promising prognosis. A small sample (*N* = 112) prospective cohort study among AKI patients at the Clinical Center Nis, Serbia suggested that serum ferritin levels on admission were higher in AKI recovery group compared with the non-recovery group (34). However, a prospective study evaluated 120 patients who underwent procedures at another tertiary referral center, serum ferritin levels did not differ in the groups with and without ARF in those patients (35).

OASIS (Oxford Acute Severity of Illness Score) (36), SAPS II (Simplified Acute Physiology Score ii) (37) and SOFA (Sequential Organ Failure Assessment Score) (38, 39) are common scores for disease assessment in ICU. APACHE ii is the most commonly used illness scoring system in ICUs today (40), but due to the wide variety of assessments, accurate data cannot be extracted from the MIMIC-IV database currently. These scoring systems are designed to focus on the patient’s vital signs, state of consciousness, and important organ function indicators, with higher scores suggesting that the patient’s condition is more severe and the likelihood of a worse prognosis (41). A prospective, multicenter, observational study included 2,954 critically ill patients with AKI in 30 intensive care units of 28 tertiary hospitals in Beijing, China, intending to evaluate the performance of four scoring systems in predicting 28-day mortality. The study showed that OASIS, APACHE II, and SAPS II had better predictive accuracy than SOFA, but due to the computational complexity of APACHE II and SAPS II, OASIS is a good alternative (42). In our study, as the value of SF increased, the OASIS, SAPSii and SOFA scores increased accordingly in both Q2 and Q3 groups, indicating that the severity of the disease was higher in these patients. The results suggest that when dealing with critically ill AKI patients, we should perform illness assessment scores such as OASIS, SAPS ii, etc., meanwhile we should also be concerned about hyperferritinaemia, which is strongly associated with a poorer prognosis, regardless of the presence of sepsis in this AKI patient.

In chronic kidney disease (CKD) patients, serum iron abnormalities were commonly occurred because of lack of erythropoietin (EPO), uremic toxins or dialysis-induced erythrocyte destruction, immune disorders and acid–base balance disturbances. These abnormalities may lead to an increased risk of death (24, 43). Many studies have attempted to find an appropriate ferritin threshold to distinguish between iron deficiency or iron overload, or to predict disease progression, but a single serum ferritin threshold cannot be appropriate for all diseases. The baseline level of SF may change if the patient has a comorbid condition such as liver disease, iron-deficiency anemia, or malignancy, but this is also a sign of a complex and serious medical condition that may lead to a worse prognosis. Therefore, it is necessary to develop corresponding thresholds for each specific condition. In this study, patients comorbid hematologic diseases, tumor, carcinoma, hemorrhage diseases, or undergoing maternity events was excluded, minimized effects of blood loss and consumptive diseases on ferritin. In our study, the Q3 group had a significantly higher HR in all death events in 90 days, which was not observed in the Q2 group, and the range of SF values in the Q3 group was >680 ng/mL. These results suggest that serum ferritin >680 ng/mL is associated with all-cause mortality in AKI patients.

## 5 Conclusion

Our study demonstrated serum ferritin elevation (SF > 680 ng/mL) was significantly associated with 28-day mortality in AKI patients, with or without sepsis, which may further facilitate the clinical application of serum ferritin as a biomarker in prognosis of AKI.

## 6 Limitations

This is a retrospective cohort study based on MIMIC-IV 2.2, a single-center clinical database. Whether the same phenomenon exists in clinical medical centers in other countries and regions requires further study. Due to the lack of SF results, quite a number of AKI patients were excluded. Erythrocyte sedimentation rate (ESR) and C-reactive protein (CRP) are well recognized acute-phase response proteins, but they were not included in this study because they had more than 25% missing values in the study population. Cystatin C, a recognized biomarker of kidney injury, were not included in the MIMIC-IV database. Further studies need to incorporate more factors to validate this result.

## Data availability statement

The datasets presented in this study can be found in online repositories. The names of the repository/repositories and accession number(s) can be found at: This is a retrospective cohort study. All data were obtained from the Medical Information Mart for Intensive Care (latest version MIMIC-IV 2.2) database (https://mimic.mit.edu/). MIMIC is a public database and contains the clinical information of critical ill patients from 2008 to 2019 who admitted in intensive care unit (ICU) of Beth Israel Deaconess Medical Center (Boston, Massachusetts). The author has passed the Protecting Human Research Participants exam and obtained the certification (certification number 38100618) for utilizing the MIMIC-IV database.

## Ethics statement

The studies were conducted in accordance with the local legislation and institutional requirements. The human samples used in this study were acquired from Establishment of the MIMIC-IV database was approved by the Massachusetts Institute of Technology (Cambridge, MA) and Beth Israel Deaconess Medical Center (Boston, MA), and consent was obtained for the original data collection. Therefore, the ethical approval statement and the need for informed consent were waived for the studies on this database. Written informed consent for participation was not required from the participants or the participants’ legal guardians/next of kin in accordance with the national legislation and institutional requirements.

## Author contributions

XR: Data curation, Investigation, Resources, Writing – original draft. ZJ: Data curation, Formal analysis, Software, Writing – review & editing. FL: Methodology, Visualization, Writing – original draft. QW: Data curation, Validation, Visualization, Writing – original draft. HC: Methodology, Visualization, Writing – review & editing. LY: Investigation, Software, Writing – review & editing. CM: Visualization, Writing – review & editing. RW: Funding acquisition, Methodology, Resources, Supervision, Writing – review & editing.

## Footnotes

^1^ https://mimic.mit.edu/
